# A Liquid Path to Lung Disease: EarlyArsenicExposureand EffectsinYoung Adults

**Published:** 2006-08

**Authors:** Tanya Tillett

Increased rates of cancer and mortality have been documented in areas of the world where drinking water contains high concentrations of naturally occurring arsenic. A new study by a group of Californian and Chilean researchers now provides strong evidence of a link in humans between prenatal and early childhood arsenic exposure and significantly higher rates of lung disease in young adulthood **[*EHP* 114:1293–1296; Smith et al.]**.

Both malignant and nonmalignant lung disease are known to develop with exposure to arsenic in drinking water. Recent evidence from a project in India by the same research group showed decreased lung function similar to that of smokers in adults exposed to the semimetallic carcinogen.

The current study took advantage of a unique opportunity to study the long-term health effects of a discrete prenatal and early childhood exposure. From 1958 to 1970, the water supply for the neighboring Chilean cities of Antofagasta and Mejillones was supplemented with water from rivers with arsenic concentrations near 1,000 μg/L, 100 times the current acceptable standard for arsenic concentration in the United States. With the 1971 activation of an arsenic removal plant, however, levels plummeted to about 90 μg/L and have continued to drop ever since.

The research team studied mortality data obtained from Chile’s Ministry of Health for the years 1989 through 2000 for all 13 regions of the country. They divided the population into two groups: individuals born between 1958 and 1970 (who likely would have had prenatal arsenic exposure if their mothers lived in Antofagasta or Mejillones) and those born between 1950 and 1957 (who likely would have had childhood but not prenatal exposure if they lived in either of the two cities). The researchers also divided overall deaths for Chile into two groups: residents of Antofagasta and Mejillones, and residents of all other regions. They used the *International Classification of Diseases, Ninth Revision,* to code causes of death, including lung cancer and bronchiectasis, a form of chronic respiratory disease.

The investigators’ findings show a distinct connection between prenatal and early childhood exposure to arsenic and lung disease–related mortality before age 50. Lung cancer death rates for those exposed to arsenic in early childhood were 7 times higher than those for the rest of the Chilean population, and bronchiectasis death rates were 12 times higher. In cases where exposure occurred both prenatally and in early childhood, lung cancer death rates were 6 times higher and bronchiectasis death rates were 46 times higher than those for the rest of the population.

The authors believe these results describe the highest increase in death rates for arsenic-related lung cancer and bronchiectasis ever documented among young adults, and add that this study is one of the first to provide evidence of human adult disease resulting from prenatal and early childhood exposure to any environmental toxicant. They conclude that an increase in young adult mortality should be of concern to public health officials, and should influence future decisions regarding sources of drinking water.

## Figures and Tables

**Figure:**
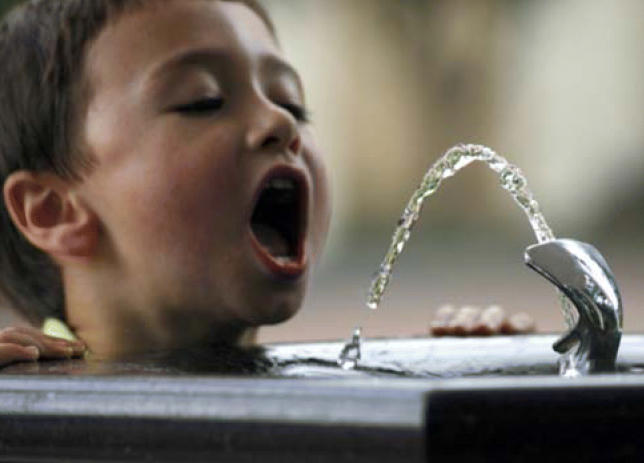
A big gulp of news The link between early arsenic exposure and later lung disease is the first such association to be confirmed in humans.

